# Case report: A balance of survival and quality of life in long-term survival case of lung adenocarcinoma with synchronous bone metastasis

**DOI:** 10.3389/fonc.2022.1045458

**Published:** 2022-10-26

**Authors:** Yao Xu, Haixiao Wu, Cong Wang, Yulin Ma, Chao Zhang

**Affiliations:** ^1^ Tianjin Medical University Cancer Institute and Hospital, National Clinical Research Center for Cancer, Key Laboratory of Cancer Prevention and Therapy, Tianjin’s Clinical Research Center for Cancer, Tianjin, China; ^2^ The Sino-Russian Joint Research Center for Bone Metastasis in Malignant Tumor, Tianjin, China

**Keywords:** lung adenocarcinoma, bone metastasis, survival outcome, quality of life, case report

## Abstract

Bone metastasis is one of the comorbidities of advanced lung cancer, eventually leading to an impaired quality of life. We present a case of a lung adenocarcinoma patient with synchronous bone metastasis. The patient possessed a superior survival time of more than five years under multidisciplinary treatment. Considering the balance of life expectancy and limb function, the metastatic site on the right humerus was successively surgically managed. Based on the present case, we emphasized the importance of treatment choice between anti-tumor and bone management in the long-term survival of cancer patients with synchronous bone metastasis.

## Introduction

Lung cancer is the leading cause of cancer mortality worldwide. In the United States, estimated deaths of lung and bronchus cancer were up to 68,820 and 61,360 for males and females, respectively, both of which accounted for around one fifth of the total cancer deaths (1). The 3-year relative survival of lung cancer has been rising from 21.0% to 31.0%, which is attributed to the improvement of tumor screening and multidisciplinary therapy (1, 2). More than half of the newly diagnosed lung cancer patients presented with metastatic disease at initial diagnosis (3). Data from the SEER program demonstrated the corresponding 5-year relative survival rate was a dismal 6.9% for lung cancer patients with distant metastasis (4).

In this study, bone was one of the most common metastatic sites in lung cancer, and bone metastasis (BM) occurred in more than one third of the advanced patients, especially in non-small cell lung cancer (NSCLC) patients (5). Due to the longer survival expectancy and the improvement of imaging technology, the reported BM incidence of lung cancer has been increasing (6). Bone metastasis and subsequent skeletal-related events (SREs) impaired activities of daily life and reduced quality of life (QOL), which eventually deteriorated the general condition and shortened the survival time of the patients (7). The previous study concluded that the median survival of lung cancer patients with synchronous BM was 4.00 (95% CI: 3.89–4.11) months (8). In our study on lung patients with synchronous BM, a total of 938 patients were selected, and the median survival of patients was up to 11.53 months (9). With the development of therapeutic modalities, the survival outcome of advanced lung cancer patients has been improving.

Skeletal-related events (SREs) are accepted to result in impaired mobility and increased mortality in cancer patients with BM. SREs are comprised of several complications, such as bone pain, pathological bone fracture, hypercalcemia, the need for radiotherapy, and spinal cord compression (10). Bone resorption inhibitors, such as bisphosphonates and denosumab, were recommended routinely. These anti-resorptive agents have been shown to delay the occurrence of SREs and reduce the frequency of SREs by regulating bone microenvironment (11). Radiation therapy (RT) was recommended for local control and pain palliation of bone metastatic sites. The dose and fractionation of RT should be tailored to the symptom and general condition of the patients (12). As for patients with pathological fractures or spinal cord compression, surgical intervention was emphasized to palliative symptoms and stabilizes bone structure. Importantly, life expectancy should be evaluated before surgery performance (13).

In this study, we reported a female lung cancer case diagnosed with synchronous BM. Accompanied with multidisciplinary management, three operations were performed on her bone metastasis site. At the last follow-up in September 2022, the patient had lived more than 5 years after diagnosis. In this case, we emphasized the importance of treatment choice between anti-tumor and bone management in the long survival of cancer patients with synchronous bone metastasis.

## Case description

**Figure 1:**
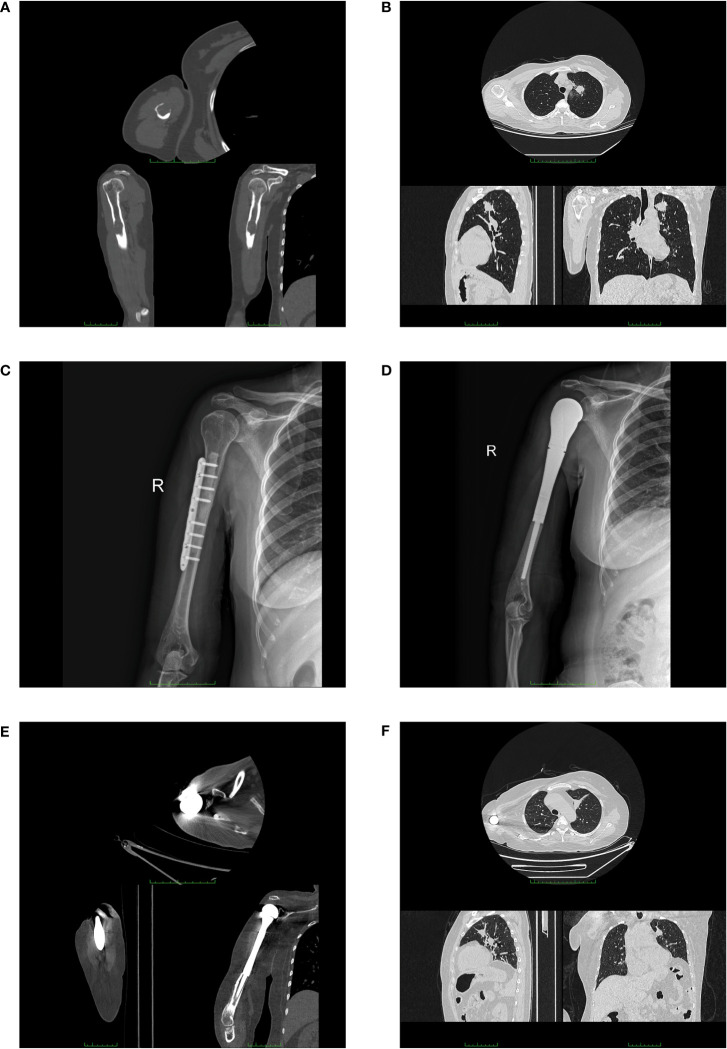
Imaging examination of the patient. Computed tomography images (upper image for axial, lower left for sagittal, and lower right for coronal, respectively) of the patient at the initial diagnosis **(A, B)**. **(A)** CT scan showed a soft tissue mass (2.4 cm × 1.8 cm) and a pathologic fracture on the right humerus of the patient. **(B)** Chest CT showed a pulmonary mass in the left upper lobe. X-ray images after the first two surgeries **(C, D)**. **(C)** X-ray image after the surgery for plate internal fixation on the right humerus. **(D)** X-ray image after the surgery for the right shoulder joint replacement. Computed tomography images (upper image for axial, lower left for sagittal, and lower right for coronal, respectively) before the surgery of shoulder disarticulation **(E, F)**. **(E)** CT scan showed the prosthesis of patient exposed out of right humerus. **(F)** Chest CT presented stable disease in the primary lung tumor.

A 52-year-old woman was admitted to our department in September 2016 due to pain (VAS = 6) in her right upper arm. The study was conducted in accordance with the Declaration of Helsinki and approved by the Ethics Board of the Tianjin Medical University Cancer Institute and Hospital. The signed informed consent of the patient was obtained. The study was reported in agreement with the principles of the CARE guidelines (14). A computed tomography (CT) scan revealed the evidence of a soft tissue mass and pathologic fracture on the right humerus (**Figure 1A**). At the same time, a pulmonary CT presented space-occupying lesions on her left upper lobe (**Figure 1B**). Based on the Mirels scoring system, the patient got a 9, which indicated a high risk of pathological fracture. Thus, the patient underwent tumor resection, bone cement filling, and plate internal fixation on the right humerus. X-ray imaging after surgery is shown in **Figure 1C**. The postoperative pathological diagnosis was metastatic adenocarcinoma (T2aN0M1b, stage IVA), and the metastatic site was considered to have originated from the lung based on immunohistochemistry testing. Pathologic imaging is presented in **Supplementary Figure 1**. After recovery from surgery, five cycles of palliative chemotherapy were carried out (pemetrexed 800 mg plus carboplatin 500 mg) until stable disease of primary lung cancer was detected by a routine imaging test, accompanied by the infusion of pamidronic acid monthly.

The patient was referred to our hospital again in September 2018 because of the recurrence at the original metastatic site and severe pain (VAS = 7). A PET-CT examination was performed in order to assess the progression of the disease. The right axilla lymph node was enlarged, being diagnosed as lymphatic metastasis. Besides, several new bone destruction sites were detected, including the previous surgical site on the right head of the humerus, the fourth lumbar, the right iliac bone, and the left femur. Thus, the patient was diagnosed with multiple bone metastases (T2aN3M1c, stage IVB). The patient got a new Mirels score of 9. After the prognostic evaluation (more than 6 months) and the general condition assessment, in order to prevent pathologic fracture of the right humerus, the replacement of the right shoulder joint was performed (**Figure 1D**). The pathological result was confirmed to be a metastatic adenocarcinoma with poor differentiation (**Supplementary Figure 2**). Two cycles of chemotherapy (pemetrexed 800 mg plus carboplatin 500 mg) were performed with no remission. Thus, a new regimen of systematic chemotherapy was needed. In March 2019, the patient underwent genetic testing of postoperative paraffin-embedding tissue and the results concluded that both the mutations of EGFR, HER2, and MET and the rearrangements of ALK and ROS1 were negative. Meanwhile, the expression level of PD-L1 was less than 1%. After multidisciplinary team discussion, the chemotherapy was performed, which comprised docetaxel 110 mg and carboplatin 500 mg. Meanwhile, sintilimab 200 mg was given at the strong will of the patient. The regimen was terminated in May 2019 due to the intolerant allergy. Then, the carboplatin was replaced by nedaplatin 140 mg, and the regimen was regularly performed for four courses.

**Figure 2:**
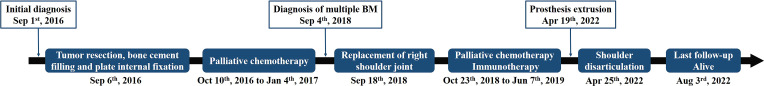
The timeline of the diagnosis and treatment course of the patient in the present case.

After an approximately three-year stable disease period, the patient presented to our department with the occurrence of prosthesis extrusion in April 2022. The CT imaging is shown in **Figure 1E**. Physical examination showed a skin ulceration (5.0 cm ∗ 2.0 cm) on the previous surgical site with swelling and exudation, accompanied by no systemic symptoms of fever, sweats, or chills. The bacterial culture test of exudation was performed, and pseudomonas aeruginosa was found. The serum examination indicated an elevation of C-reactive protein (52.22 mg/L) without other abnormalities. Considering the unsatisfactory expected function of the limb and the poor general condition (Karnofsky score = 50) of the patient, the surgery of right shoulder disarticulation was performed with the consent of the patient. Early functional exercise and mental rehabilitation were applied after surgery. The patient is still in follow-up with stable disease in his primary lung tumor after long-term systematic therapy (**Figure 1F**). The timeline of the diagnosis and treatment course of the patient is summarized in **Figure 2**.

## Discussion

With the advance of targeted therapy and immunotherapy in recent years, new medications such as tyrosine kinase inhibitors (TKIs) and anti-PD1/PD-L1 agents have been approved and widely used in patients with advanced lung cancer (2). We reviewed the previous literature reporting long-term survival in lung cancer patients with synchronous BM and listed four case reports in **Table 1** (15–18). Most studies were conducted before the application of targeted therapy. The treatment was restricted to conventional therapies such as systematic chemotherapy, surgical resection, and radiotherapy, which seemed to be largely out-of-data in current treatment principles. Hou et al. reported a 76-year-old female lung cancer patient presenting with metastasis on the first lumbar vertebra and the left seventh anterior rib at the initial diagnosis. The patient underwent surgery for lumbar vertebra tumor removal, bone cement filling, and pedicle screw fixation at the spine metastatic site. After surgery, the backache symptoms of the patient were significantly relieved. Two cycles of chemotherapy followed by EGFR-TKI geftinib were performed and the patient achieved an eight-year survival. In the present case, we also presented a female lung cancer patient with long-term survival. In contrast to the spinal metastatic case reported by Hou et al., our patient suffered from intolerable pain and immobility due to the metastasis on the right humerus. In the absence of individual comprehensive therapy, systematic treatment was performed to achieve a longer survival outcome. Meanwhile, the preservation and restoration of limb function in BM patients should be pursued after evaluating the balance of cancer survival and quality of life.

**Table 1:** Summary of the previously reported long-term survival (more than five years) cases of lung cancer patients with synchronous bone metastasis.

Publication year	Age at initial diagnosis	Gender	Pathology	Primary lung tumor treatment and systematic therapy	Bone metastatic site	Bone metastatic site treatment	Survival time
2021 (15)	76	Female	Papillary adenocarcinoma, moderately differentiated	Two cycles of chemotherapy; EGFR-TKI geftinib	1^st^ lumbar vertebra and left 7^th^ anterior rib	Lumbar vertebra tumor removal, bone cement flling and plate internal fxation; radiation therapy; zoledronic acid intravenous injection	Eight years survival after initial diagnosis
2005 (16)	71	Male	Squamous cell carcinoma, well differentiated	Left lower lobe lobectomy plus six cycles of systematic chemotherapy; radiation therapy after local recurrence	Right fibula	None	Five years survival after last operation
	52	Male	Adenocarcinoma, poorly differentiated	Right upper lobe lobectomy plus three cycles of systematic chemotherapy	Left thigh	Resection of the left thigh tumor	Five years survival after last operation
2005 (17)	61	Male	NSCLC, poorly differentiated	Chemotherapy plus radiation to the left upper lobe tumor	Right femur	Right femoral intramedullary rod placement plus radiation therapy; excision of the right femoral metastasis after recurrence	Eight years survival after initial diagnosis
2004 (18)	68	Male	Adenocarcinoma	Left upper lobectomy and resection of partial parietal pleura	Right iliac bone	Resection of the right iliac bone tumor	Five years survival after initial diagnosis

**Figure 3:**
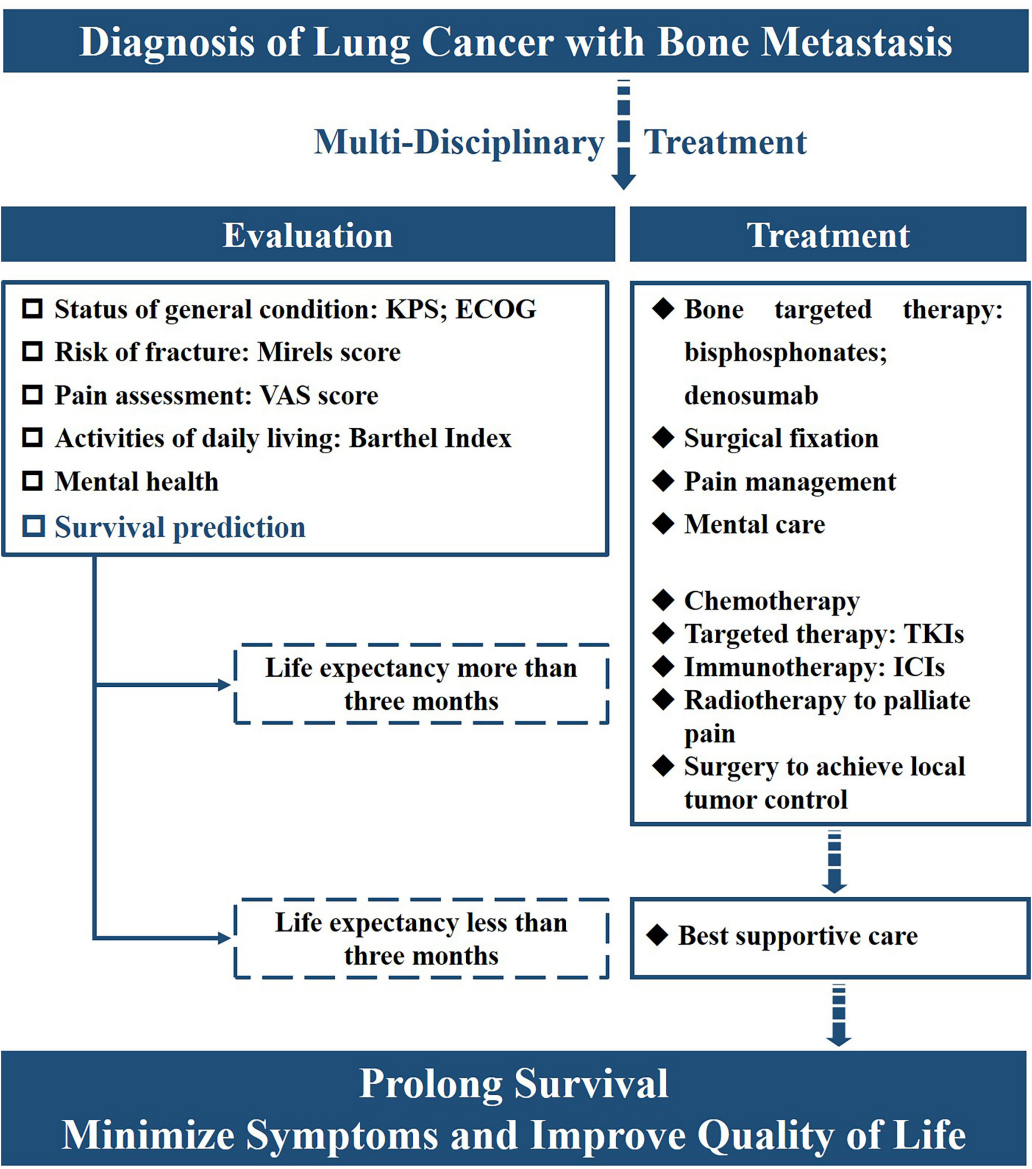
A flowchart illustrating comprehensive evaluation and treatment strategies for lung patients with bone metastasis.

As shown in **Figure 3**, the goal of treatment for advanced-stage lung cancer is to achieve local control, minimize symptoms, and improve QOL (19). The multidisciplinary approach conducted by the team of oncologists, orthopedic surgeons, radiologists, pathologists, psychologists, and palliative medicine specialists should be emphasized before the accurate prediction of survival. Several prognostic models were reported to predict the survival of lung patients with BM, which played an important role in treatment decision-making (8, 20–22). For NSCLC patients with a predictive life expectancy of less than three months, best supportive care was recommended, which aimed to reduce suffering and support the best possible QOL (23). For BM patients with a survival time of more than 3 months, systematic therapy including bone-targeted therapy, radiotherapy, and pain management should be performed. Meanwhile, surgical intervention was recommended for patients with a survival time of more than 6 months (12). To identify targeted therapy or immunotherapy potentially benefiting survival, molecular testing should be performed and individual treatment should be planned according to the resting results (12). Notably, systemic imaging examination was emphasized for advanced cancer patients to detect unusual metastatic sites (24). Solitary suspicious lesions, especially those in uncommon locations such as the skull, should be carefully diagnosed to exclude metabolic and other diseases (25). The therapeutic regimen must be initiated under the circumstances of the right diagnosis.

The exposure of the prosthesis was one of the infrequent complications of joint replacement. Michala et al. reported a total of 130 patients who received joint replacement due to skeletal metastasis, and one case presented a prosthesis penetrating the skin (26). In the current study, the body mass index (BMI) of the patient decreased from 24.0 to 19.2 since the performance of shoulder joint replacement. Besides, the patient suffered from an impact on her right shoulder, which might have led to the rupture of the fragile fiber formed after surgery. Thus, we suspected that the prosthesis exposure was attributed to malnutrition and the experience of falling over. After the comprehensive discussion on the general condition, comorbidity, and economic condition of the patient, the patient chose to have disarticulation surgery. Before the disarticulation surgery, the infectious situation could not be satisfactorily controlled with the antibiotics, which were associated with the immunosuppression after long-term anti-immune treatment. The infection could increase the probability of allograft rejection, leading to the failure of revision surgery. Besides, two-time surgeries had been performed, which reduced the remaining soft tissue to cover the surgical site and increased the operational difficulty of revision surgery. Thus, disarticulation surgery, with fewer postoperative complications and a lower cost, was a more suitable choice for the patient than revision surgery.

The patient queried for the surgery of shoulder disarticulation autonomously, which reflected the pursuit of the patient for improvement of QOL. For an advanced lung cancer patient with BM, the demand of patient at initial diagnosis was cancer control and survival extension. After several invasive methods and subsequently systematic treatment, local control was achieved temporarily, which was always accompanied by cancer-associated complications and treatment-related adverse events (27). Systematic chemotherapy might cause liver dysfunction and myelosuppression alone or in combination with immunotherapy (28). It was reported that immune-related adverse effects such as thyroid disorders occurred in NSCLC patients who were receiving immunotherapy (29). As for patients undertaking surgical intervention, some of them suffered from perioperative and postoperative complications, which included pneumonia, infectious diseases, and neurological deterioration (29). Besides, the financial burden on patients and their families sustainably accumulated, which caused distress in patients and finally turned their demands from longer survival to palliating symptoms and improving QOL (30). Thus, cancer itself as well as social factors should be focused on, which urgently needs support offered by psychological consultants and medical social workers (10). The clinical management of BM was one of the major concerns worldwide (31). It was reported that the major complications of BM commonly occur within 3–6 months in patients without the treatment of bone resorption inhibitors (32). Thus, it is important to initiate a bone management program as soon as the diagnosis of BM is made, regardless of whether the patient presents SREs or not (10). Bone resorption inhibitors such as bisphosphonates and denosumab were highly recommended after the diagnosis of BM, which aimed to prevent or delay the occurrence of SREs (33). To palliate symptom of pain, oral drugs such as NSAIDs and opioid treatment and radiation therapy were effective approaches. Several options for radiation therapy are currently available, including external-beam radiation therapy (EBRT), stereotactic body irradiation therapy (SBRT), and radionuclide treatment (11).

In the current case, the patient was diagnosed with a solitary bone metastasis on the right humerus, and a surgical metastectomy was performed initially. With the development of cancer, oligometastatic disease (with a limited number of metastatic sites) was identified. Since the occurrence of disease progression, local surgical intervention was performed to prevent pathologic fracture and systemic treatment was conducted, all of which were chosen after comprehensive evaluation of survival and limb function. As for patients with oligometastatic bone disease (OMBD), the local therapy of both primary lung cancer and bone metastatic sites should be highlighted. In a meta-analysis including 757 patients with oligometastatic NSCLC, prolonged survival was observed in patients who received consolidative local treatment (34). The aggressive surgical metastectomy and radiotherapy on bone metastasis may benefit survival in oligometastatic NSCLC patients (35, 36). Since complete pathologic fractures are a clear indication for surgery, the selected patients with impending fractures should undergo surgical intervention to prevent fractures. According to the NCCN guidelines, orthopedic stabilization accompanied by palliative radiotherapy should be performed in patients as risk of fracture on weight-bearing bone (12). Meanwhile, a previous study concluded that patients with impending pathologic fractures had a better outcome than those with complete fractures after surgical intervention (37). Thus, the optimal time to perform surgery for patients with impending fractures needs further investigation after the consideration of the benefit from surgery. As shown in **Table 1**, various cases suggested cancer patients could significantly benefit from the multidisciplinary treatment, even if they were diagnosed with the initial advanced stage. Thus, a multidisciplinary approach should be encouraged, and the necessary psychological counseling should be given to such patients. Currently, rarely is a study looking into the individualized needs of cancer patients with BM performed. With improved survival of cancer patients with BM, such a study would be of significance for guiding treatment plan making. At the same time, more attention should be given to the field of precise prognostication in cancer patients with BM.

In summary, we described a case of lung adenocarcinoma in the left upper lobe, diagnosed with synchronous bone metastasis. With more than 5 years of survival since the initial diagnosis, the patients obtained survival benefits from the current systematic chemotherapy and targeted therapy. Meanwhile, the limb function of the patient was reserved as much as possible despite the multiple recurrences until the final disarticulation. The study aimed to propose the concept that treatment modalities should be tailored to the life expectancy and the individual needs of patients.

## Data availability statement

The original contributions presented in the study are included in the article/**Supplementary Material**. Further inquiries can be directed to the corresponding author.

## Ethics statement

This study was reviewed and approved by the Tianjin Medical University Cancer Institute and Hospital. The patients/participants provided their written informed consent to participate in this study. Written informed consent was obtained from the individual(s) for the publication of any potentially identifiable images or data included in this article.

## Author contributions

YM and CZ contributed to the conception and design of the study. YX wrote the first draft of the manuscript. HW wrote some sections of the manuscript. CW made a substantial contribution to the acquisition and interpretation of the data for the work. All authors contributed to the article and approved the submitted version.

## Conflict of interest

The authors declare that the research was conducted in the absence of any commercial or financial relationships that could be construed as a potential conflict of interest..

## Publisher’s note

All claims expressed in this article are solely those of the authors and do not necessarily represent those of their affiliated organizations, or those of the publisher, the editors and the reviewers. Any product that may be evaluated in this article, or claim that may be made by its manufacturer, is not guaranteed or endorsed by the publisher.
