# Supplementary figures and images for: Case report: A balance of survival and quality of life in long-term survival case of lung adenocarcinoma with synchronous bone metastasis
Source: Front Oncol. 2022 Oct 26;12:1045458. doi: 10.3389/fonc.2022.1045458 (PMC9644070; doi:10.3389/fonc.2022.1045458)

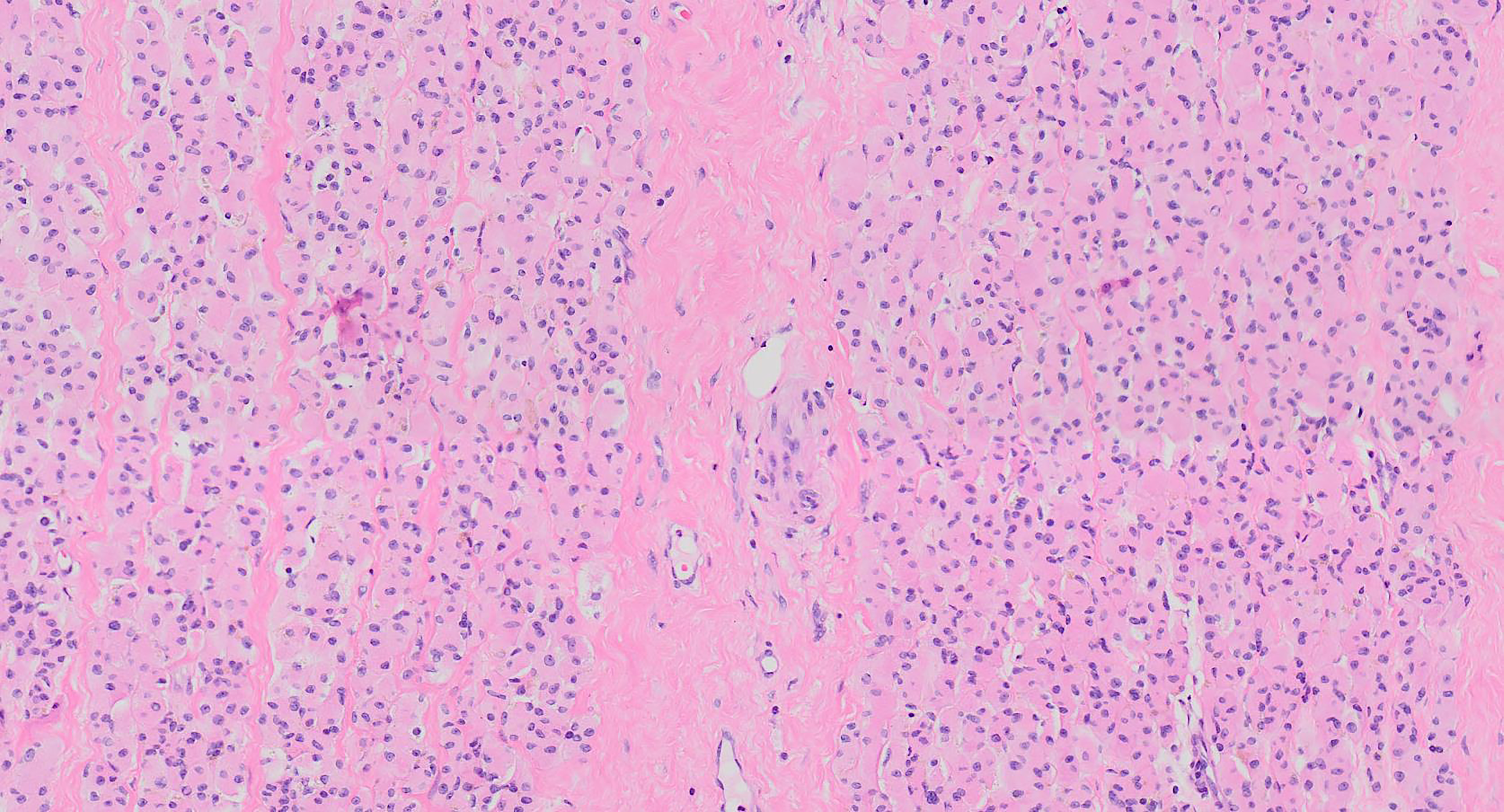

Supplement: Supplementary file 1 [file Image_1.tif]

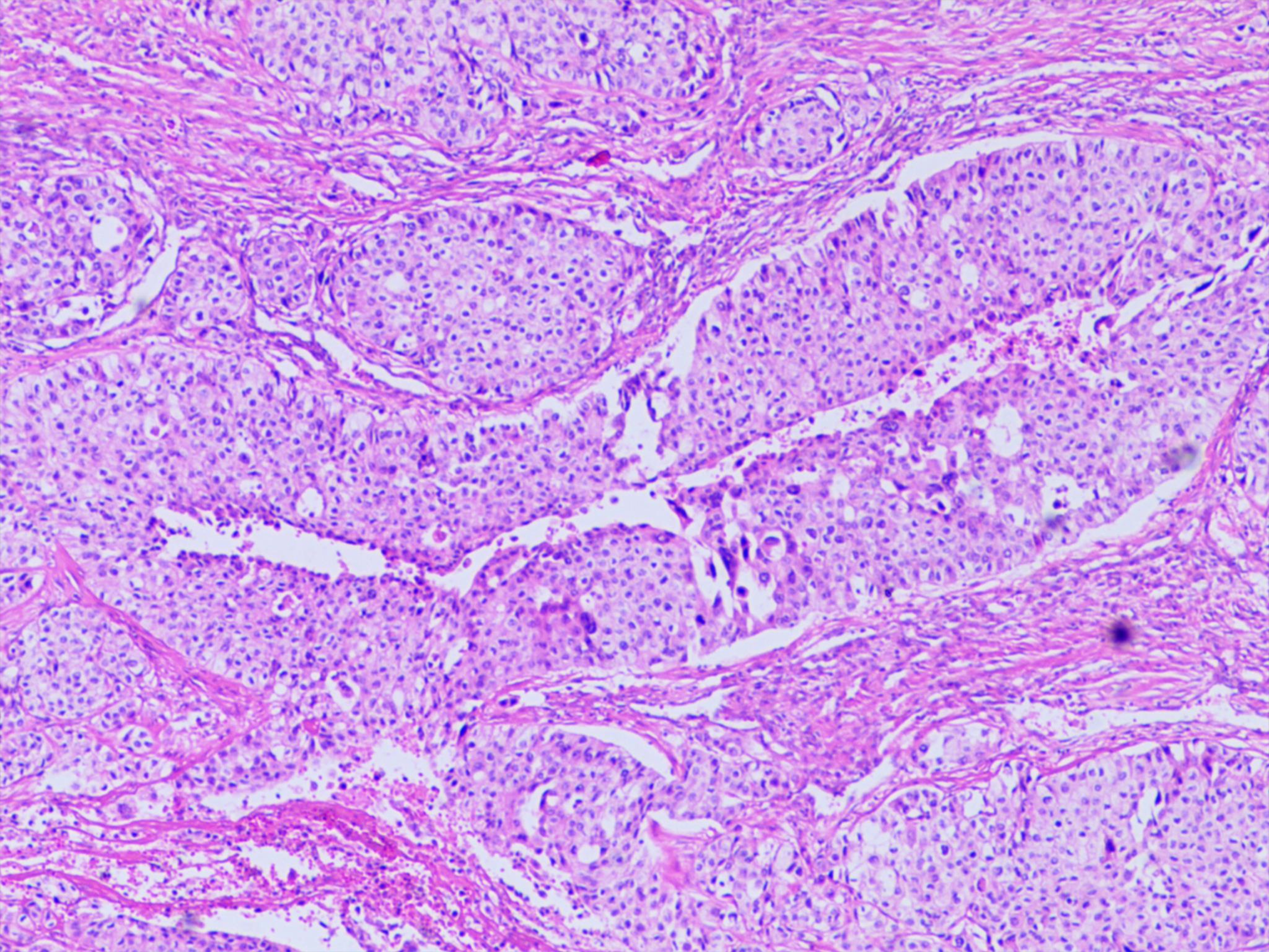

Supplement: Supplementary file 2 [file Image_2.tif]
